# Endosymbiont *Tremblaya phenacola* influences the reproduction of cotton mealybugs by regulating the mechanistic target of rapamycin pathway

**DOI:** 10.1093/ismejo/wrae052

**Published:** 2024-03-22

**Authors:** Jianyang Bai, Zhangqi Zuo, Haonan DuanMu, Meizhen Li, Haojie Tong, Yang Mei, Yiqi Xiao, Kang He, Mingxing Jiang, Shuping Wang, Fei Li

**Affiliations:** State Key Laboratory of Rice Biology & Ministry of Agriculture and Rural Affairs Key Laboratory of Molecular Biology of Crop Pathogens and Insects, Institute of Insect Sciences, College of Agriculture and Biotechnology, Zhejiang University, Hangzhou 310058, China; State Key Laboratory of Rice Biology & Ministry of Agriculture and Rural Affairs Key Laboratory of Molecular Biology of Crop Pathogens and Insects, Institute of Insect Sciences, College of Agriculture and Biotechnology, Zhejiang University, Hangzhou 310058, China; State Key Laboratory of Rice Biology & Ministry of Agriculture and Rural Affairs Key Laboratory of Molecular Biology of Crop Pathogens and Insects, Institute of Insect Sciences, College of Agriculture and Biotechnology, Zhejiang University, Hangzhou 310058, China; State Key Laboratory of Rice Biology & Ministry of Agriculture and Rural Affairs Key Laboratory of Molecular Biology of Crop Pathogens and Insects, Institute of Insect Sciences, College of Agriculture and Biotechnology, Zhejiang University, Hangzhou 310058, China; State Key Laboratory of Rice Biology & Ministry of Agriculture and Rural Affairs Key Laboratory of Molecular Biology of Crop Pathogens and Insects, Institute of Insect Sciences, College of Agriculture and Biotechnology, Zhejiang University, Hangzhou 310058, China; State Key Laboratory of Rice Biology & Ministry of Agriculture and Rural Affairs Key Laboratory of Molecular Biology of Crop Pathogens and Insects, Institute of Insect Sciences, College of Agriculture and Biotechnology, Zhejiang University, Hangzhou 310058, China; State Key Laboratory of Rice Biology & Ministry of Agriculture and Rural Affairs Key Laboratory of Molecular Biology of Crop Pathogens and Insects, Institute of Insect Sciences, College of Agriculture and Biotechnology, Zhejiang University, Hangzhou 310058, China; State Key Laboratory of Rice Biology & Ministry of Agriculture and Rural Affairs Key Laboratory of Molecular Biology of Crop Pathogens and Insects, Institute of Insect Sciences, College of Agriculture and Biotechnology, Zhejiang University, Hangzhou 310058, China; State Key Laboratory of Rice Biology & Ministry of Agriculture and Rural Affairs Key Laboratory of Molecular Biology of Crop Pathogens and Insects, Institute of Insect Sciences, College of Agriculture and Biotechnology, Zhejiang University, Hangzhou 310058, China; Technical Centre for Animal, Plant & Food Inspection and Quarantine, Shanghai Customs, Shanghai 200135, China; State Key Laboratory of Rice Biology & Ministry of Agriculture and Rural Affairs Key Laboratory of Molecular Biology of Crop Pathogens and Insects, Institute of Insect Sciences, College of Agriculture and Biotechnology, Zhejiang University, Hangzhou 310058, China

**Keywords:** Phenacoccus solenopsis, endosymbiont, nutrition compensation, fecundity, coevolution

## Abstract

The intricate evolutionary dynamics of endosymbiotic relationships result in unique characteristics among the genomes of symbionts, which profoundly influence host insect phenotypes. Here, we investigated an endosymbiotic system in *Phenacoccus solenopsis*, a notorious pest of the subfamily Phenacoccinae. The endosymbiont, “*Candidatus* Tremblaya phenacola” (*T. phenacola* PSOL), persisted throughout the complete life cycle of female hosts and was more active during oviposition, whereas there was a significant decline in abundance after pupation in males. Genome sequencing yielded an endosymbiont genome of 221.1 kb in size, comprising seven contigs and originating from a chimeric arrangement between betaproteobacteria and gammaproteobacteria. A comprehensive analysis of amino acid metabolic pathways demonstrated complementarity between the host and endosymbiont metabolism. Elimination of *T. phenacola* PSOL through antibiotic treatment significantly decreased *P. solenopsis* fecundity. Weighted gene coexpression network analysis demonstrated a correlation between genes associated with essential amino acid synthesis and those associated with host meiosis and oocyte maturation. Moreover, altering endosymbiont abundance activated the host mechanistic target of rapamycin pathway, suggesting that changes in the amino acid abundance affected the host reproductive capabilities via this signal pathway. Taken together, these findings demonstrate a mechanism by which the endosymbiont *T. phenacola* PSOL contributed to high fecundity in *P. solenopsis* and provide new insights into nutritional compensation and coevolution of the endosymbiotic system.

## Introduction

Nutritional symbioses between animals and microorganisms contribute positively to the adaptability of both [1]. Some symbionts play vital roles in enabling the colonization of nutrient-deficient environments through the supplementation of the host’s metabolic requirements, which makes insects thrive on imbalanced carbohydrate-based diets [2, 3]. Long-term symbiotic relationships shape the bacterial genome and significantly affect the host phenotypes related to nutrition, metabolism, reproduction, immunity, and development [4–7]. For example, a genomic research on the pea aphid, *Acyrthosiphon pisum*, revealed an extensive metabolic exchange with its obligate nutritional endosymbiont, *Buchnera aphidicola* [8]. Such nutritional symbionts in aphids broaden the dietary range, influencing reproduction processes during aphids’ sexual phase [9]. Another interesting study of carpenter ant and its primary endosymbiont, *Blochmannia floridanus*, showed that the symbiont provides essential nitrogen and sulfur compounds to the ants, benefiting in return from the ants’ metabolic processes [10]. The intricate symbiotic partnerships between hosts and their endosymbionts lead to highly specialized interactions that can significantly affect the biology, ecology, and evolution of both partners.

Mealybugs (Hemiptera: Coccoidea: Pseudococcidae) are recognized as invasive pests in numerous regions across the world [11]. These diminutive sap-sucking insects feed on a broad range of vegetables, horticultural plants, and field crops, causing huge economic losses worldwide [12, 13]. Previous studies have identified two subfamilies of mealybugs, Pseudococcinae and Phenacoccinae [14], which have distinct lineages of bacterial endosymbionts [15] and patterns of nutritional complementation. A representative pest of the Phenacoccinae subfamily, cotton mealybug (*Phenacoccus solenopsis* Tinsley), infests over 200 plant species and has been observed in at least 35 geographic regions worldwide [16, 17]. The broad adaptability of this pest has been attributed in part to its beneficial mutualistic endosymbiotic system.

Mealybug primary endosymbionts (P-endosymbionts) are maternally inherited and localize to specialized host cells known as bacteriocytes. These cells accumulate to form larger structures, bacteriomes [18, 19]. Subfamily Pseudococcinae P-endosymbionts belong to the betaproteobacteria class “*Candidatus* Tremblaya princeps,” (*T. phenacola* PSOL), which in turn host co-obligate gammaproteobacterial endosymbionts [20]. This three-way symbiotic system has been extensively studied in terms of horizontal gene transfer, metabolic patchwork, and coevolution [18, 21–23]. By contrast, members of the subfamily Phenacoccinae contain only the betaproteobacterium “*Candidatus* Tremblaya phenacola” [24]. The sequencing and analysis of the genome of *T. phenacola* PPER (isolated from *Phenacoccus peruvianus*) revealed genome fusion between a betaproteobacterium and a gammaproteobacterium [25]. Furthermore, phylogenetic studies of *T. phenacola* and other insects within the subfamily Phenacoccinae have suggested independent symbiont coevolution in each insect host species [26, 27].

In addition to essential amino acids synthesis, mutualistic symbiosis in insects are capable of producing some important molecules that affect specific biological process. A recent study on aphid-*Buchnera* symbiosis highlights the differential expression of various eukaryotic cell signaling pathways in bacteriocytes of low-titer versus high-titer hosts. In low-titer genotypes, cell-growth pathways are upregulated, while in high-titer genotypes, pathways related to membrane trafficking, lysosomal processes, the mechanistic target of rapamycin (mTOR), and cytokine pathways are more active [28]. The mTOR signaling pathway is implicated in regulating both the reproductive capacity and lifespan of social insects, particularly termites [29]. The influence of essential amino acids produced via the mutualistic system on the reproductive processes of the cotton mealybug continues to be an area of uncertainty.

To gain a better understanding of coevolution and mutualistic symbiosis between the cotton mealybug and *Tremblaya*, we here examined the distribution of *T. phenacola* PSOL in male and female cotton mealybugs. We then assembled the *T. phenacola* PSOL genome to analyze the phylogenetic relationships among *Tremblaya* symbionts. Finally, we analyzed metabolic complementation within the symbiotic system and assessed the impacts of this system on cotton mealybug fecundity. These experiments directly demonstrate endosymbiont impacts on host insect reproductive phenotypes, providing deepened insights into the coevolution of an agriculturally important insect pest and its bacterial endosymbiont.

## Materials and methods

### Insect population

A laboratory colony of cotton mealybugs was used for these experiments. The progenitors were originally collected from the ornamental plant Rose of Sharon (*Hibiscus syriacus* L.) in Jinhua, China. Insects were reared on tomato plants (*Solanum lycopersicum*) in a climate-controlled chamber at 27 ± 1°C with 75% relative humidity.

### Fluorescence in situ hybridization

To detect the distribution of the endosymbiont in the cotton mealybug, we used Fluorescence *in Situ* Hybridization (FISH), employed in a previous study [30]. In details, the second and third instar larvae, female adult, pupa, and male adult of cotton mealybugs were disinfected using 70% alcohol for 1 min, followed by 0.01% sodium hypochlorite for 1 min. They were then rinsed with sterile ddH_2_O three times. The samples were fixed in Carnoy’s solution (chloroform: ethanol: acetic acid = 6:3:1) at 55°C for 30 min. After removing the fixatives, the samples were washed three times in sterile phosphate-buffered saline (PBS) buffer, then placed in 6% H_2_O_2_-ethanol solution to decolorize for 1 h. After the decolorizing agent was removed, hybridization solution (HYBA) Buffer (comprising 5 ml methanamide, 2.5 ml 20× saline sodium citrate, 100 μl heparin, 100 μl 10% Tween-20, and 105 μl 100 μg/ml salmon sperm DNA) was added to each sample for prehybridizing at 65°C for 2 h. The specific 16S rDNA probe (cy5–5’ATCTACGCATTTCACCGCTACTCCTGGAATTCTATCCCCCTCTTCCATACTCGAG-3′) was mixed with HYBA Buffer at a ratio of 1:99 and then incubated for 5 min at 80°C; 1 ml HYBA Buffer/probe mixture was then added to each sample and was incubated overnight at 65°C in the dark. Samples were washed with PBS + Tween, then incubated with 4′,6-diamidino-2-phenylindole for 30 min. Each sample was then observed and photographed on an LSM 800 laser confocal microscope (ZEISS, Oberkochen, Germany).

### Bacteriome sequencing and assembly

About 150 female mealybugs were starved for 48 h, then the bacteriomes of these mealybugs were collected for *T. phenacola* PSOL DNA extraction. In detail, individuals were disinfected in 70% alcohol for 1 min followed by 0.01% sodium hypochlorite for 1 min; rinsed with sterile ddH_2_O three times; then rinsed with sterile 0.2 M PBS (pH 6.8) three times. Bacteriomes were collected through insect dissection in a drop of sterile 0.2 M PBS in a sterile petri dish (1.5 × 9 cm) under an SMZ 645 stereo microscope (Nikon, Tokyo, Japan). The collected bacteriomes were gently washed twice with sterile 0.2 M PBS prior to DNA extraction with a DNA Isolation Mini Kit (Vazyme Biotech, Nanjing, China).

Extracted *T. phenacola* PSOL DNA was used to generate paired-end Illumina libraries with a 350-bp insert size. Libraries were sequenced on the HiSeq X-Ten platform (Illumina, San Diego, CA, USA). Duplicate reads were removed with FastUniq v1.1 [31] and the remaining reads were merged with FLASH v1.2.11 [32]. Merged reads were first mapped to the cotton mealybug genome assembly [33] with BWA v0.7.17 [34], followed by the filtration of host DNA contamination using Samtools v1.17 [35]. The whole-body PacBio reads of the cotton mealybug (PRJNA380754) were used as queries in BLAST searches against the cotton mealybug genome assembly using Megablast v2.14.0+ [36]. Unaligned sequences were collected with Seqkit v2.4.0 [37] and combined with the unmapped Illumina short reads; these sequences were regarded as candidate symbiont reads. A hybrid assembly was conducted in Unicycler using the “normal” mode [38]. Contigs of <500 bp in size were excluded from further analyses. The final assembly coverage was calculated with Samtools v1.17 [35].

### *T. phenacola* PSOL genome annotation and taxonomic assignment

The *T. phenacola* PSOL genome was annotated to identify protein-coding, transfer RNA, and ribosomal RNA (rRNA) genes. These were predicted using the RAST server v1.1.0 [39] with default parameters and an *E*-value of 1e-5. The information on repetitive sequences was predicted using the RepeatMasker web server (https://www.repeatmasker.org/cgi-bin/WEBRepeatMasker). Conserved syntenic blocks between *T. phenacola* PSOL and *T. phenacola* PAVE or *T. phenacola* PPER were identified with TBLASTX and visualized in Circos [40].

Taxonomic assignments were conducted using two methods. First, all predicted protein-coding genes from *T. phenacola* PSOL were used as queries in BLAST searches against the NCBI nonredundant protein sequence (nr) database with the parameters “-evalue 1e-5 -outfmt 5.” The output xml file was then analyzed in Megan v6.12.2 [41]. Second, phylogenetic gene trees were constructed for each *T. phenacola* PSOL gene to determine the putative evolutionary position. A betaproteobacteria-gammaproteobacteria reference database was constructed from the protein sequences of all sequenced *Tremblaya* symbiovars, their obligatory gammaproteobacteria, and 24 489 betaproteobacterial and 122 778 gammaproteobacterial protein sequences downloaded from UniProtKB. Protein sequences of the alphaproteobacterium *Rickettsia* were downloaded from UniProtKB and used as an outgroup. For each *T. phenacola* PSOL protein, a phylogenetic tree was constructed from the 30 best BLASTP hits against the beta–gamma database, the two best BLASTP hits against the alpha database, and the target protein itself. Protein sequences were aligned by MAFFT v7.310 [42]. The program trimAL v1.2rev59 [43] with the “-automated1” parameter was used to exclude ambiguously aligned positions. The best substitution model was selected using Bayesian Information Criterion in IQ-TREE v1.5.5 [44] with the “test” mode. IQ-TREE was then used to construct a tree for each protein using the maximum likelihood (ML) method with 1000 bootstrap replicates. Single-gene trees were visualized by ggtree v2.4.0 [45]. Based on these steps, we finally obtained 92 beta-origin and 104 gamma-origin genes in *T. phenacola* PSOL.

### Phylogenetic analyses of different mealybugs and their symbionts

Five mealybug genome assemblies were downloaded from the European Nucleotide Archive: *Maconellicoccus hirsutus* (PRJEB12066), *Ferrisia virgata* (PRJEB12067), *Pseudococcus longispinus* (PRJEB12068), *Paracoccus marginatus* (PRJEB12069), and *Trionymus perrisii* (PRJEB12071). These genomes were annotated using combined evidence from Augustus [46], BLAST, and HMMER [47]. The aphid *A. pisum* (GenBank accession GCA_000142985.2) was used as the outgroup. Proteins encoded by each gene family were aligned and trimmed as described above for single-gene tree construction. All of the trimmed single-copy protein sequences were then concatenated to generate one super-gene for each species. An ML tree was constructed based on the super-gene with the JTT + F + I + G4 method and 1000 bootstrap replicates.

For each class of proteobacteria, several mealybug symbionts and representative proteobacteria were selected for analysis. The *T. phenacola* PSOL and *T. phenacola* PPER proteins of betaproteobacterial and gammaproteobacterial origin (referred to as beta-origin and gamma-origin genes, respectively) were used in betaproteobacteria and gammaproteobacteria phylogenetic tree constructions, respectively. The alphaproteobacterium *Rickettsia prowazekii* (RefSeq accession GCF_000195735.1) was used as the outgroup. Phylogenetic trees were constructed as described above, with the betaproteobacterial tree based on 28 single-copy proteins (Table S1) using the LG + I + G4 model and the gammaproteobacterial tree based on 23 single-copy proteins (Table S2) using the Q.yeast+I + G4 model.

### Substitution rate analysis

Single-copy beta-origin orthologs between *T. phenacola* PSOL and *T. princeps* PCIT and single-copy gamma-origin orthologs between *T. phenacola* PSOL and *Moranella endobia* PCIT were aligned with MAFFT v7.310. KaKs Calculator v2.0 [48] was employed to calculate the nonsynonymous substitution (*dN*) to synonymous substitution (*dS*) ratio between orthologous pairs with the parameters “-c 11 -m MA.” We also calculated the *dN*/*dS* ratio between orthologous pairs of *T. phenacola* PSOL and *T. phenacola* PPER using the same method. All statistical analyses and visualizations were performed in R v3.5.2.

### Amino acid biosynthesis pathway construction

Genes involved in amino acid biosynthesis pathways were identified using an annotated cotton mealybug genome (http://v2.insect-genome.com/Organism/624), the *T. phenacola* PSOL genome annotated here, and the BlastKOALA tool on the Kyoto Encyclopedia of Genes and Genomes (KEGG) website (https://www.genome.jp/kegg/) [49]. Similarly, we analyzed the amino acid biosynthesis pathways of the other five mealybugs including *F. virgata*, *M. hirsutus*, *P. longispinus*, *P. marginatus*, *T. perrisii*. These genome data were obtained from a previous study in 2016 [18]. Complete metabolic pathways in these mealybug symbiotic systems were constructed using KEGG Mapper (https://www.genome.jp/kegg/mapper/) [50].

### Mealybug phenotype analysis

Mealybugs were treated with antibiotics to reduce endosymbiont abundance. Tetracycline and gentamicin were obtained from Solarbio Technology Co., Ltd (Beijing, China). Antibiotic solutions (0.25 mg/ml and 0.75 mg/ml for both antibiotics) were prepared in sterile ddH_2_O. Three-week-old tomato plants were positioned such that the branch roots were inside plastic cups (5 × 7 × 8 cm) filled with 20 ml of antibiotic solution or ddH_2_O as a control. Tomato branches were vertically secured within the plastic cups using foam trays (0.4 × 5 cm thickness × diameter). Antibiotic solution was refreshed every 48 h until insects reached adulthood. The durations of preoviposition, oviposition, and postoviposition were recorded, as were the number of offspring and the offspring sex ratios. Each group contained 14–32 female adults, with 1 female representing one biological replicate.

To evaluate the physiological impact of antibiotics on mealybugs, we evaluated the effects of different antibiotic concentrations on mealybug survival rates, and each group contained 45 mealybug individuals. We also measured the impact of antibiotics on the ATPase content in cotton mealybugs using insect-ATPase ELISA kit (Kenuodibio, China). To process the insect samples for biochemical analysis, we homogenized 50 mg mealybugs for each group in 500 μl of ice-cold PBS. The homogenates were then centrifuged at 1000 × *g* for 20 min, and the supernatant was collected for further analysis. For the construction of a standard curve, we prepared dilutions of a standard substance to concentrations of 0.5, 1, 2, 4, 8, and 16 U. In a 96-well plate, we added 50 μl of the supernatant, followed by 100 μl of horseradish peroxidase-conjugated detection antibody, and it was incubated at 37°C for 60 min. After incubation, the liquid was discarded, and the plate was washed five times with washing buffer. Subsequently, 50 μl each of substrates A and B were added, and the plate was incubated at 37°C for 15 min. The reaction was stopped by adding 50 μl of stop solution, and the absorbance was measured at a wavelength of 450 nm.

Levels of free amino acids were tested in the control group and in the 0.25 mg/ml tetracycline group of mealybugs. Briefly, a collection of mealybugs from each treatment group weighing 50 mg in total were placed in separate centrifuge tubes and were homogenized in 500 μl of 2% sulfosalicylic acid. The homogenates were passed through a 0.22-μm filter membrane, then the free amino acid concentrations of each solution were measured with an L8900 amino acid analyzer (HITACHI, Tokyo, Japan). The data were visualized in GraphPad Prism 9.0.

### RNA sequencing and gene expression analysis

First, second, third, pupa, male, and female of the cotton mealybugs were collected for RNA extraction. For each sample, the collection included 50 individuals for the group of first, second, and pupa; 100 individuals for male adults; 15 individuals for third and female adults. Total RNA was extracted from each replicate sample using TRIzol reagent (Thermo Fisher Scientific, Waltham, MA, USA). Sequencing libraries were constructed using an NEBNext Ultra RNA Library Prep Kit (New England Biolabs, Ipswich, MA, USA). All experiments were repeated in triplicate. In detail, mRNA enrichment was initially carried out using Oligo(dT) magnetic beads, followed by the fragmentation of mRNA. Single-stranded cDNA was then synthesized using random hexamer primers. Subsequently, double-stranded cDNA was produced by adding buffer, dNTPs, DNA polymerase I, and RNase H, then purified using AMPure XP beads. Finally, the double-stranded cDNA was processed for end repair, poly-A tailing, and adapter ligation, preparing it for sequencing. The resulting libraries were sequenced on the HiSeq4000 platform (Illumina) to generate paired-end 150-bp reads. Raw reads were filtered using fastp v0.23.4 [51] and then mapped to reference genome of cotton mealybug using HISAT v2.2.1 [52]. StringTie v2.2.1 [53] was used to assemble transcripts and estimate their abundance.

Gene expression levels were calculated in fragments per kilobase of transcript per million mapped reads. Based on these values, a gene coexpression network was constructed with weighted gene coexpression network analysis (WGCNA) [54, 55]. The TOMSimilarity module was used to calculate the coexpression similarity coefficients between genes with the following parameters: power = 6, TOMType = unsigned, deepSplit = 2, mergeCutHeight = 0.25, and numericLabels = TRUE. KEGG enrichment analysis of each cluster was performed with clusterProfiler v4.6.2 [56].

### Quantitative reverse transcription

Total RNA of the cotton mealybugs was extracted using TRIzol reagent, and 1 μg of RNA was reverse-transcribed using HiScript III 1^st^ Strand cDNA Synthesis Kit (Vazyme Biotech). We designed gene-specific primers based on the sequence of 16S rRNA of *T. phenacola* PSOL for endosymbiont quantification, with the host’s *Actin* selected as the reference gene (Table S3). Quantitative reverse transcription was performed on a QuantStudio thermocycler (Thermo Fisher Scientific) in 20-μl reaction volumes containing 10 μl of HiScript III RT SuperMix (Vazyme), 1 μl of 100 ng/μl cDNA, and 1 μl of 50 ng/μl primers. The thermocycling program was as follows: initial denaturation, 95°C for 10 min; 40 cycles of denaturation (95°C for 15 s) and annealing (60°C for 30 s). The data were analyzed using the 2^−ΔΔC^ method [57]. These experiments were conducted with at least three independent biological replicates.

### Statistical analyses

Differences between treatment groups in the preoviposition duration, postoviposition duration, and number of offspring were assessed with a Poisson regression or Poisson-like regression model. A logistic regression model was used to examine treatment-based differences in the sex ratio. Differences between treatment groups in other parameters were assessed with a one-way analysis of variance (ANOVA).

## Results

### Dynamic distribution of *T. phenacola* PSOL in cotton mealybugs

**Figure 1:**
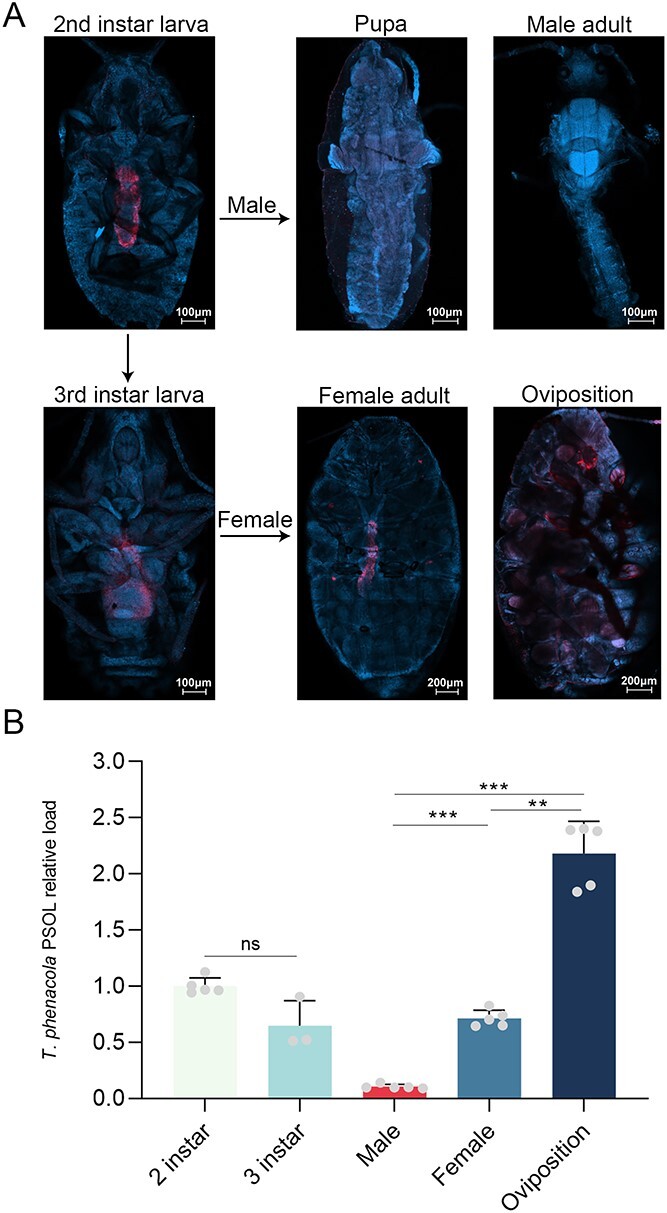
Dynamic distribution of *T. phenacola* PSOL in cotton mealybugs; (A) distribution of *T. phenacola* PSOL in different developmental stages of cotton mealybug; (B) the relative quantification of *T. phenacola* PSOL in cotton mealybugs, and actin served as the reference gene; data were analyzed by Tukey’s multiple comparisons test (^**^P < .01, ^***^P < .001).

We first conducted FISH experiments to detect the endosymbiont distribution within the host using a *T. phenacola* PSOL-specific 16S rDNA probe. In female mealybugs, the endosymbiont was present in the bacteriome of the abdominal midsection throughout the entire lifecycle (Fig. 1A). By contrast, the bacteriome gradually disintegrated during pupation in males, resulting in a faint, dispersed signal in adult males (Fig. 1A). Subsequent qPCR experiments revealed significant differences in the abundance of *T. phenacola* PSOL between female and male mealybugs (Fig. 1B, *P* < .001). These results were comparable, with consistent levels throughout the lifecycle in the females and sharply decreased abundance in adult males. During the oviposition process in female mealybugs, we observed a significant increase in the abundance of *T. phenacola* PSOL (Fig. 1B, *P* < .01).

### *T. phenacola* PSOL genome analysis reveals a fusion of beta and gamma proteobacterial genes

**Figure 2:**
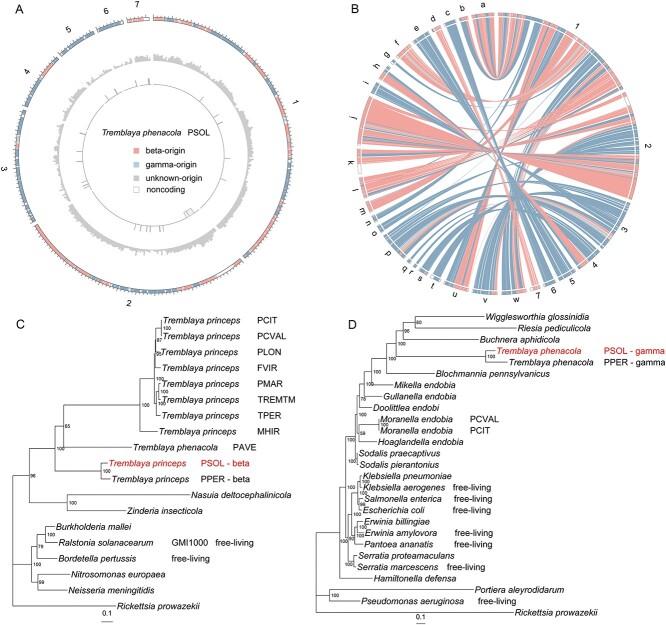
Genome characteristics and evolutionary analysis of *T. phenacola* PSOL; (A) genome assembly of *T. phenacola* PSOL; the denotation of each track is as follows (from outside to the inside): seven contigs of *T. phenacola* PSOL genome; GC-content; repeat sequence; (B) syntenic blocks between *T. phenacola* PSOL and *T. phenacola* PPER; (C) phylogenetic tree of beta-proteobacteria and the beta-proportions of *T. phenacola* PSOL and PPER; (D) phylogenetic tree of gamma-proteobacteria and the gamma-proportions of *T. phenacola PSOL* and PPER; *T. phenacola* PSOL are colored in red.

In a previous study, our lab isolated four scaffolds originating from *T. phenacola* PSOL using a PacBio library of DNA extracted from cotton mealybug [33]. To improve the endosymbiont genome assembly, we extracted bacteriome DNA to sequence *T. phenacola* PSOL directly*.* A total of 6.44 Gb clean data from *T. phenacola* PSOL bacteriomes were obtained, yielding a high-quality assembly comprising seven contigs with a genome size of 221.1 kb with 100 × coverage (Fig. 2A). The new genome assembly included 229 predicted protein-coding genes. Single-gene phylogenetic trees and analysis with Megan indicated that these genes had three distinct origins: betaproteobacteria (40.2%), gammaproteobacteria (45.4%), and unknown (14.4%) (Fig. 2A). Both beta-origin and gamma-origin genes of *T. phenacola* PSOL exhibited a high synteny with *T. phenacola* PPER (*E*-value <1e-5, identity >75%, length > 100 bp) (Fig. 2B), and most of the beta-origin genes also displayed high synteny with the *T. phenacola* PAVE genome (Fig. S1). Compared with other known *Tremblaya* genomes, *T. phenacola* PSOL has the largest genome size but the lowest GC content (34.8%) (Table S4). Compared with *T. phenacola* PPER, *T. phenacola* PSOL possesses a higher number of genes originating from gamma sources (Fig. S2). This difference might contribute to the lower GC content observed in *T. phenacola* PSOL. KEGG analysis indicated that beta-origin and gamma-origin genes generally had roles in distinct biological processes. For example, most genes predicted to be involved in environmental adaptation, transcription, and nucleotide metabolism are of beta-origin, whereas those involved in immune system and cell motility were gamma-origin genes (Fig. S3).

To elucidate the evolutionary relationships among mealybug endosymbionts, a phylogenetic tree was constructed. The *T. phenacola* PSOL was most closely related to *T. phenacola* PPER, whereas *T. phenacola* PAVE was more closely related to *T. princeps* symbiovars (Fig. 2C). The gammaproteobacteria present in *T. phenacola* PSOL and those isolated from *T. phenacola* PPER clustered together within the same monophyletic group, which was a sister taxon of the *Sodalis* lineage (Fig. 2D).

### Genes originating from both beta and gammaproteobacteria are evolutionarily conserved

**Figure 3:**
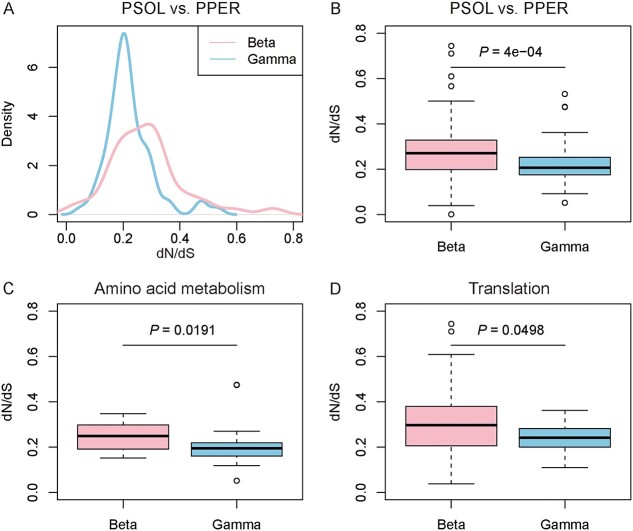
Rates of synonymous (*dS*) and nonsynonymous (*dN*) substitutions between orthologous genes from the same lineage in *T. phenacola* PSOL and *T. phenacola* PPER; (A) density distribution of *dN*/*dS* rates for beta-origin and gamma-origin genes; (B) *dN*/*dS* rates of beta-origin genes and gamma-origin genes (Wilcoxon rank sum test, *P* = 4e-4); (C) *dN*/*dS* rates of beta-origin genes involved in amino acid metabolism categories (*P* = .0191); (D) *dN*/*dS* rates of gamma-origin genes involved in translation categories (*P* = .0498).

To evaluate the selective strength of genes in *T. phenacola* PSOL, we calculated the rates of synonymous (*dS*) and nonsynonymous (*dN*) substitutions between orthologous genes from the same lineage (beta or gamma) in *T. phenacola* PSOL and *T. phenacola* PPER. All *T. phenacola* PSOL genes had a *dN*/*dS* rate < 1 (mean = 0.20), but gamma-origin genes had significantly lower *dN*/*dS* rates than beta-origin genes (Wilcoxon rank sum test, *P* = 4 × 10^−4^) (Fig. 3A and B). For amino acid metabolism and translation processes, gamma-origin genes are under less selective pressure (Fig. 3C and D). Similar results were also obtained from substitution analysis in *T. phenacola* PSOL and the symbiotic system of *T. princeps* PCIT (beta) and *M. endobia* PCIT (gamma) (Fig. S4). Besides, gene expression analysis revealed that gamma-origin genes, especially those related to amino acid synthesis, are highly expressed during the female adult stage (Fig. S5B and Table S5).

### Metabolic complementation for amino acid synthesis

**Figure 4:**
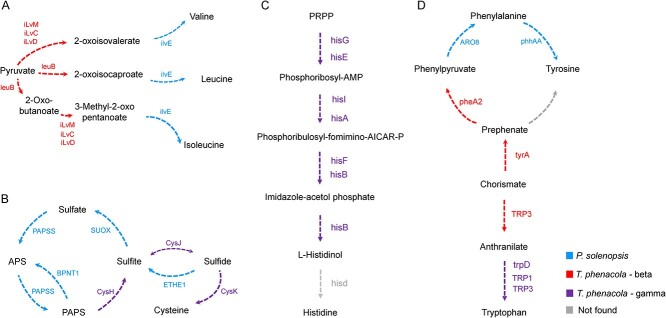
Complementary amino acid synthesis pathways of the cotton mealybug and *T. phenacola* PSOL; (A) the patterns diagram of valine, leucine, and isoleucine synthesis; (B) cysteine synthesis; (C) histidine synthesis; (D) phenylalanine, tyrosine, and tryptophan synthesis.

Given high conservation of *T. phenacola* PSOL in the amino acid metabolism pathway and its nutritional complementation with the host, we constructed amino acid biosynthesis pathways using protein-coding genes from both cotton mealybug and the endosymbiont. The results indicated that the two species formed highly complementary “patchwork” metabolic pathways (Fig. 4), with varying components of each pathway present in only one partner. Endosymbiont genes participating in valine, leucine, and isoleucine biosynthesis were of betaproteobacterial origin (Fig. 4A), whereas genes involved in cysteine and histidine biosynthesis were of gammaproteobacterial origin (Fig. 4B and C). Other genes (such as those involved in tryptophan, tyrosine, and phenylalanine biosynthesis) were of both betaproteobacterial and gammaproteobacterial origin (Fig. 4D). Besides, upon comparing the amino acid synthesis pathways of various symbiotic systems, we observed that these pathways are largely conserved, yet exhibit species-specific characteristics across different mutualistic systems (Fig. S6). Compared to other systems, the genes for amino acid synthesis enzymes show less redundancy in *T. phenacola* PSOL (Fig. S6B).

### *T. phenacola* PSOL promotes cotton mealybug fecundity

**Figure 5:**
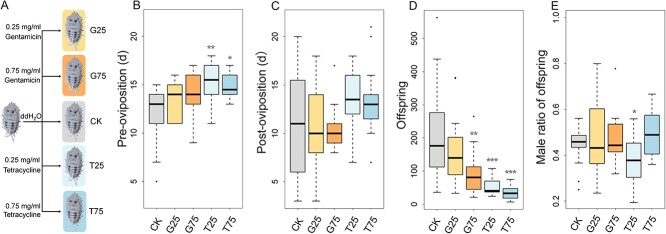
Reproductive phenotype determination of the cotton mealybug after interfering *T. phenacola* PSOL with antibiotic; (A) construction of antibiotic interference model with tetracycline and gentamicin; (B) the duration between sexual maturity and the onset of spawning in the cotton mealybugs from different groups; (C) total spawning time of the cotton mealybugs from different groups; (D) number of offspring in different groups; (E) male ratio of offspring in different groups; the dots represent individual discrete values; each group contains 14–32 female adults, and each female adult represents a repeat all data of reproduction were analyzed with Poisson regression, and male ratio was analyzed with logistic regression; ^*^P < .05, ^**^P < .01, and ^***^P < .001.

To gain deep insights into the effects of amino acids produced by this patchwork on insect physiology, the cotton mealybugs were exposed to antibiotics (Fig. 5A). Due to the inevitable physiological impact of antibiotics on insects, we assessed the effects of antibiotic toxicity on cotton mealybug survival rates and mitochondrial ATPase activity. Our results indicated that both gentamicin and tetracycline had no significant impact on these aspects (Fig. S7A and D). Treatment with tetracycline significantly inhibited the abundance of *T. phenacola* PSOL (Fig. S7B), leading to a decrease in partial free amino acid contents (Fig. S7C) and even impacting wax synthesis (Fig. S7E).

The preoviposition period was significantly extended in groups treated with 0.25 mg/ml or 0.75 mg/ml tetracycline (*P* < .05; Poisson regression) (Fig. 5B). Because oocysts persist in female adults upon death, the oviposition period and the postoviposition period were combined into a single period (postoviposition) for further analysis; this period was also extended in females treated with tetracycline (Fig. 5C). Female insects treated with either the low or high concentration of tetracycline showed a significant reduction in the number of offspring produced (Fig. 5D, *P* < .001; Poisson-like regression). Treatment with 0.25 mg/ml tetracycline led to a decline in the proportion of male offspring (*P* < .05; logistic regression), but no other antibiotic treatment significantly influenced the offspring sex ratio (Fig. 5E).

### *T. phenacola* PSOL activated the host mechanistic target of rapamycin pathway

**Figure 6:**
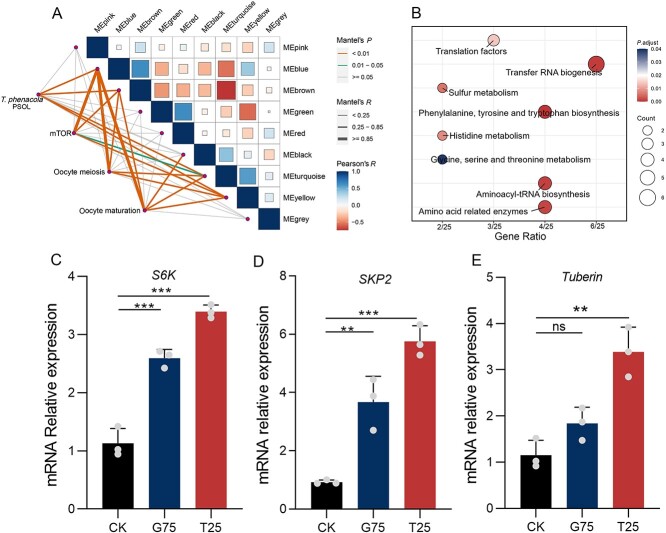
Gene coexpression network analysis based on reproductive phenotype; (A) correlation diagrams between different modules clustered by WGCNA; the hub genes in the MEblue module were utilized to create network diagrams composed of multiple line segments; (B) KEGG enrichment analysis of the genes from *T. phenacola* PSOL in the MEblue module; (C)–(E) represent the relative expression of key genes involved in mTOR signal pathway after interfering the *T. phenacola* PSOL with 0.75 mg/ml gentamicin and 0.25 mg/ml tetracycline; data were analyzed with one-way ANOVA test; ^**^P < .01, ^***^P < .001, and ns represents no significant difference among the groups.

To further explore the mechanism by which the endosymbiont regulated host reproduction, we conducted a comprehensive gene coexpression analysis. Hierarchical clustering and pruning of adjacent dissimilarity yielded a gene-dependent dendrogram (Fig. S8A). The blue module exhibited a strong correlation with female developmental stage (Fig. S8B). Furthermore, genes in this module exhibited significant correlations between expression in *T. phenacola* PSOL and in several cotton mealybug processes, including the mTOR signaling pathway, oocyte meiosis, and oocyte maturation (Fig. 6A). KEGG enrichment analysis of endosymbiont genes in the blue module revealed a notable enrichment of genes associated with the amino acid synthesis pathway (Fig. 6B). To assess whether the endosymbiont mediates mTOR signaling through essential amino acid synthesis, thereby influencing host oocyte meiosis and maturation, we analyzed the expression levels of three genes (*S6K*, *SKP2*, and *Tuberin*) in this pathway enriched in the blue module. The results showed that antibiotic treatment significantly activated the mTOR pathway of the cotton mealybug (Fig. 6C–E).

## Discussion

Many insects harbor symbionts that supply them with essential nutrients, providing benefits in development, immune metabolism, and various other biological processes [58–62]. In return, insects provide nutrient-rich intracellular niches for symbionts to colonize. Due to their extensive adaptation to the intracellular environment, nearly all endosymbionts are currently classified as unculturable. Thus, bacteriome genomic sequencing has emerged as a powerful tool to understand endosymbionts. Here, we conducted a comprehensive analysis revealing the vital role of the endosymbiont *T. phenacola* PSOL in the reproductive processes of its host, *P. solenopsis*.

We examined the distribution of *T. phenacola* PSOL across various developmental stages of the host. Our findings revealed a marked disparity in the presence of *T. phenacola* PSOL between female and male adults (Fig. 1). Similar results have also been reported in several Pseudococcinae [63]. Symbionts exhibit increased activity during oviposition (Fig. 1). This appears to be a mutual adaptation mechanism for both the symbiont and its host. In this process, symbionts enhance their chances of transmitting genetic material to offspring, whereas the host benefits from the nutrients, resulting in the production of a greater number of offspring (Figs 4 and 5). This dynamic relationship was also observed in the symbiotic system of the cereal weevil *Sitophilus oryzae*, and its midgut endosymbionts, *Sodalis pierantonius*. Following the adult metamorphosis of its host, the population of *S. pierantonius* significantly increases, but then rapidly declines and it is completely eliminated once the host finishes its cuticle synthesis [64].

In pursuit of comprehending this intriguing adaptation mechanism in the cotton mealybugs, we constructed a genome assembly of the endosymbiont *T. phenacola* PSOL (Fig. 2A). Due to limitations in sequencing methods and the repetitive sequences in the flanks (Fig. 2A), this assembly did not result in a single, complete contig for the endosymbiont. The synteny analysis with *T. phenacola* PPER indicated that this assembly is not the result of contamination or misassembly (Fig. 2B). Phylogenetic analysis indicated that *T. phenacola* PSOL was a chimeric endosymbiont resulting from genome fusion between betaproteobacteria and gammaproteobacteria. This is distinct from all other sequenced *Tremblaya* symbiovars, with the exception of *T. phenacola* PPER (Fig. 2C and D). Further analysis of the selective pressure on genes originating from betaproteobacteria and gammaproteobacteria indicates they have undergone purifying selection, with genes from gamma-proteobacteria showing a higher degree of conservation, particularly in amino acid metabolism and translation processes (Fig. 3). This is consistent with prior studies showing that a stable host environment, high nutritional supply, and redundancy with host genes lead to relaxed purifying selection within the symbiont genome [65].

Similar to other mealybug endosymbionts, the primary advantage of *T. phenacola* PSOL symbiosis to the host appeared to be provision of essential amino acids [23, 66]. Our findings here revealed a pronounced complementarity between host and endosymbiont amino acid synthesis genes, with limited functional redundancy identified between the symbiont and host genomes (Fig. 4 and Fig. S6). For example, cotton mealybug lost the cysK gene (a member of the cysteine biosynthesis pathway), but this loss was offset by the presence of a gamma-origin gene in *T. phenacola* PSOL (Fig. 4B). This stands in contrast to the *M. hirsutus* symbiotic system, which has lost the cysK gene entirely during the process of coevolution [18]. Additionally, beta-origin and gamma-origin symbiont genes in this pathway exhibited total complementarity (i.e. a complete lack of functional redundancy) (Fig. 4 and Fig. S6B). Previous studies have revealed similarly complementary patterns of gene loss and retention among the primary symbiont *Tremblaya*, the secondary symbiont *Moranella*, and the citrus mealybug host (*Planococcus citri*) [21, 67]. Overall, these data allowed us to observe the ways in which symbionts evolve in response to their own transmission, simultaneously assisting their host in overcoming adverse factors.

Mutualistic symbionts usually exert a positive effect on host’s reproduction. We here conducted the investigation of the reproductive impacts of a mutualistic endosymbiont, *T. phenacola* PSOL, on the cotton mealybug. Due to the inevitable physiological impact of antibiotics on insects, along with their suppressive effect on nonintracellular symbionts, we opted for an antibiotic with minimal intracellular action, gentamicin, and an antibiotic capable of killing intracellular bacteria, tetracycline [68, 69]. Unexpectedly, high concentrations of gentamicin still exhibited an inhibitory effect on *T. phenacola* PSOL (Fig. S7B). At the meanwhile, antibiotic interference led to reproductive disorders in the host, manifested as an extended preoviposition period and a notable decrease in offspring numbers (Fig. 5B–D). Groups treated with 0.25 mg/ml or 0.75 mg/ml gentamicin or with 0.75 mg/ml tetracycline exhibited nearly identical proportions of male offspring as untreated insects. This suggested that the presence of *T. phenacola* PSOL may not have been a significant factor in determining the sex ratio of cotton mealybug. However, several prior studies have demonstrated that nutritional bacteriocyte symbionts can manipulate the host sex ratio [61, 62, 70]. Our results underscore the considerable variation in reproductive impacts induced by different endosymbionts.

To further investigate the mechanisms through which amino acid compensation by an endosymbiont can positively impact host reproduction, we conducted a comprehensive transcriptomic analysis of the endosymbiont and the cotton mealybug at several developmental stages. WGCNA revealed one coexpression module containing *T. phenacola* PSOL genes that were coexpressed with host genes associated with amino acid biosynthesis, the mTOR pathway, and oocyte meiosis and maturation (Fig. 6A and B). This was consistent with prior studies showing that amino acids are crucial for animal reproductive processes [71–73]. Furthermore, branched-chain amino acids (namely leucine, L-isoleucine, and valine) are essential amino acids for mammals, and these metabolites activate the mTOR signal pathway through a variety of mechanisms [74]. In the present study, *T. phenacola* PSOL removal from cotton mealybug through antibiotic treatment upregulated several key genes in the mTOR pathway. One such gene was *S6K*, which is typically activated by growth factors, cytokines, and nutrients [75]. The stimulation of *S6K* in controlling cell growth has also been observed in *Drosophila* after amino acid deprivation [76]. Two other mTOR genes here found to be upregulated after antibiotic treatment, *SKP2* and *Tuberin*, have multifaceted roles in cell cycle regulation, with primary roles in the regulation of protein synthesis [77, 78]. The expression patterns of these genes reflect a strong correlation between *T. phenacola* PSOL and the mTOR pathway of host, suggesting that the endosymbiont has essential roles in influencing the host reproductive phenotype. We reason that the cotton mealybug endosymbiont influenced host reproduction by regulating the expression of genes in the mTOR pathway.

**Figure 7:**
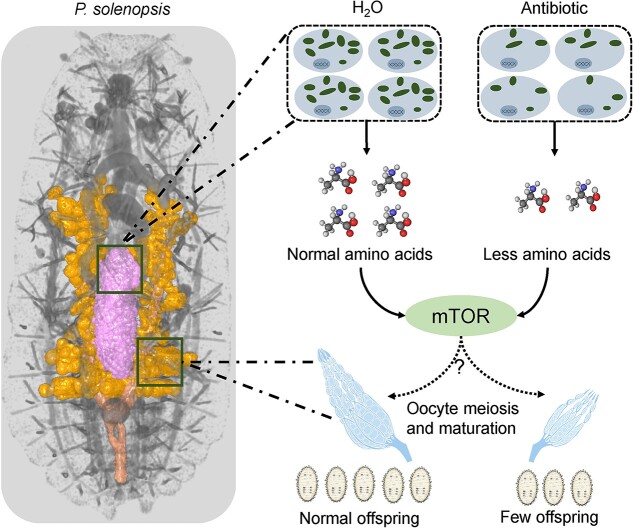
Schematic overview of how the endosymbiont, *T. phenacola* PSOL drives the cotton mealybug reproduction.

In summary, we here generated a high-quality genomic resource that not only facilitated a comprehensive investigation of the cotton mealybug genomic landscape but will also serve as a valuable reference to dissect coevolution among members of an important symbiotic relationship. Investigation of the intricate dynamics by which the endosymbiont contributes to host reproduction sheds light on the interplay between their complementary modes of nutrient acquisition (Fig. 7). Our findings underscore broader trends in mutualistic symbiotic systems across diverse ecological niches. This study thus contributes to a deep comprehension of the remarkable diversity and intricacy inherent in mealybug symbiotic relationships, opening new avenues for the ecological and evolutionary understanding of endosymbiotic systems.

## Supplementary Material

Table_S1_wrae052

Table_S2_wrae052

Table_S3_wrae052

Table_S4_wrae052

Table_S5_wrae052

Supplementary_figure_wrae052

## Data Availability

Raw sequencing reads of mealybug bacteriomes have been deposited into the NCBI (Bio-Project: PRJNA550279). The genome assembly, genome annotation, and protein FASTA files have been deposited in http://v2.insect-genome.com/Organism/624.

## References

[1] Moran NA . Symbiosis. Curr Bio*l* 2006;16:R866–71. 10.1016/j.cub.2006.09.019 17055966

[2] Heddi A , Grenier AM, Khatchadourian C et al. Four intracellular genomes direct weevil biology: nuclear, mitochondrial, principal endosymbiont, and *Wolbachia*. Proc Natl Acad Sci US*A* 1999;96:6814–9. 10.1073/pnas.96.12.6814 10359795 PMC21998

[3] Douglas AE . Phloem-sap feeding by animals: problems and solutions. J Exp Bo*t* 2006;57:747–54. 10.1093/jxb/erj067 16449374

[4] Clark EL , Karley AJ, Hubbard SF. Insect endosymbionts: Manipulators of insect herbivore trophic interactions? Protoplasm*a* 2010;244:25–51. 10.1007/s00709-010-0156-2 20495935

[5] Onchuru TO, Javier Martinez A, Ingham CS et al. Transmission of mutualistic bacteria in social and gregarious insects. Curr Opin Insect Sc*i* 2018;28:50–8. 10.1016/j.cois.2018.05.002 30551767

[6] Lee JB , Park KE, Lee SA et al. Gut symbiotic bacteria stimulate insect growth and egg production by modulating hexamerin and vitellogenin gene expression. Dev Comp Immuno*l* 2017;69:12–22. 10.1016/j.dci.2016.11.019 27932027

[7] Wilson AC , Duncan RP. Signatures of host/symbiont genome coevolution in insect nutritional endosymbioses. Proc Natl Acad Sci US*A* 2015;112:10255–61. 10.1073/pnas.1423305112 26039986 PMC4547219

[8] International Aphid Genomics C . Genome sequence of the pea aphid *Acyrthosiphon pisum*. PLoS Bio*l* 2010;8:e1000313. 10.1371/journal.pbio.1000313 20186266 PMC2826372

[9] Simon JC , Boutin S, Tsuchida T et al. Facultative symbiont infections affect aphid reproduction. PLoS On*e* 2011;6:e21831. 10.1371/journal.pone.0021831 21818272 PMC3144876

[10] Gil R , Silva FJ, Zientz E et al. The genome sequence of *Blochmannia floridanus*: comparative analysis of reduced genomes. Proc Natl Acad Sci US*A* 2003;100:9388–93. 10.1073/pnas.1533499100 12886019 PMC170928

[11] Izraeli Y , Lalzar M, Netanel N et al. *Wolbachia* influence on the fitness of *Anagyrus vladimiri* (Hymenoptera: Encyrtidae), a bio-control agent of mealybugs. Pest Manag Sc*i* 2021;77:1023–34. 10.1002/ps.6117 33002324

[12] Tong H , Wang Y, Wang S et al. Fatty acyl-CoA reductase influences wax biosynthesis in the cotton mealybug, *Phenacoccus solenopsis* Tinsley. Commun Bio*l* 2022;5:1108. 10.1038/s42003-022-03956-y 36261606 PMC9582030

[13] Arya SK , Singh S, Upadhyay SK et al. RNAi-based gene silencing in *Phenacoccus solenopsis* and its validation by in planta expression of a double-stranded RNA. Pest Manag Sc*i* 2021;77:1796–805. 10.1002/ps.6204 33270964

[14] Hardy NB , Gullan PJ, Hodgson CJ. A subfamily-level classification of mealybugs (Hemiptera: Pseudococcidae) based on integrated molecular and morphological data. Syst Entomo*l* 2010;33:51–71. 10.1111/j.1365-3113.2007.00408.x

[15] Koga R , Nikoh N, Matsuura Y et al. Mealybugs with distinct endosymbiotic systems living on the same host plant. FEMS Microbiol Eco*l* 2013;83:93–100. 10.1111/j.1574-6941.2012.01450.x 22809388

[16] Abd El-Ghany NM , Zhou JJ, Dewer Y. Antennal sensory structures of *Phenacoccus solenopsis* (Hemiptera: Pseudococcidae). Front Zoo*l* 2022;19:33. 10.1186/s12983-022-00479-4 36517816 PMC9753239

[17] Garcia , Morales M, Denno BD, Miller DR et al. ScaleNet: a literature-based model of scale insect biology and systematics. Databas*e* 2016;bav118. 10.1093/database/bav118 26861659 PMC4747323

[18] Husnik F , McCutcheon JP. Repeated replacement of an intrabacterial symbiont in the tripartite nested mealybug symbiosis. Proc Natl Acad Sci US*A* 2016;113:E5416–24. 10.1073/pnas.1603910113 27573819 PMC5027413

[19] Baumann P . Biology bacteriocyte-associated endosymbionts of plant sap-sucking insects. Ann Rev Microbio*l* 2005;59:155–89. 10.1146/annurev.micro.59.030804.121041 16153167

[20] von Dohlen CD , Kohler S, Alsop ST et al. Mealybug beta-proteobacterial endosymbionts contain gamma-proteobacterial symbionts. Natur*e* 2001;412:433–6. 10.1038/35086563 11473316

[21] Husnik F , Nikoh N, Koga R et al. Horizontal gene transfer from diverse bacteria to an insect genome enables a tripartite nested mealybug symbiosis. Cel*l* 2013;153:1567–78. 10.1016/j.cell.2013.05.040 23791183

[22] Lopez-Madrigal S , Beltra A, Resurreccion S et al. Molecular evidence for ongoing complementarity and horizontal gene transfer in endosymbiotic systems of mealybugs. Front Microbio*l* 2014;5:449 25206351 10.3389/fmicb.2014.00449 PMC4144094

[23] Szabo G , Schulz F, Toenshoff ER et al. Convergent patterns in the evolution of mealybug symbioses involving different intrabacterial symbionts. ISME *J* 2017;11:715–26. 10.1038/ismej.2016.148 27983719 PMC5322300

[24] Gruwell ME , Hardy NB, Gullan PJ et al. Evolutionary relationships among primary endosymbionts of the mealybug subfamily phenacoccinae (hemiptera: Coccoidea: Pseudococcidae). Appl Environ Micro*b* 2010;76:7521–5. 10.1128/AEM.01354-10 PMC2976180 20851962

[25] Gil R , Vargas-Chavez C, Lopez-Madrigal S et al. *Tremblaya phenacola* PPER: an evolutionary beta-gammaproteobacterium collage. ISME *J* 2018;12:124–35. 10.1038/ismej.2017.144 28914880 PMC5739004

[26] Lin D , Zhang L, Shao W et al. Phylogenetic analyses and characteristics of the microbiomes from five mealybugs (Hemiptera: Pseudococcidae). Ecol Evo*l* 2019;9:1972–84. 10.1002/ece3.4889 30847086 PMC6392364

[27] Michalik A , Michalik K, Grzywacz B et al. Molecular characterization, ultrastructure, and transovarial transmission of *Tremblaya phenacola* in six mealybugs of the Phenacoccinae subfamily (Insecta, Hemiptera, Coccomorpha). Protoplasm*a* 2019;256:1597–608. 10.1007/s00709-019-01405-y 31250115 PMC6820616

[28] Smith TE , Moran NA. Coordination of host and symbiont gene expression reveals a metabolic tug-of-war between aphids and *Buchnera*. Proc Natl Acad Sci US*A* 2020;117:2113–21. 10.1073/pnas.1916748117 31964845 PMC6995025

[29] Lin S , Werle J, Korb J. Transcriptomic analyses of the termite, Cryptotermes secundus, reveal a gene network underlying a long lifespan and high fecundity. Commun Bio*l* 2021;4:384. 10.1038/s42003-021-01892-x 33753888 PMC7985136

[30] Ye X , Xu L, Li X et al. miR-34 modulates wing polyphenism in planthopper. PLoS Gene*t* 2019;15:e1008235. 10.1371/journal.pgen.1008235 31242182 PMC6615638

[31] Xu H , Luo X, Qian J et al. FastUniq: a fast de novo duplicates removal tool for paired short reads. PLoS On*e* 2012;7:e52249. 10.1371/journal.pone.0052249 23284954 PMC3527383

[32] Magoc T , Salzberg SL. FLASH: fast length adjustment of short reads to improve genome assemblies. Bioinformatic*s* 2011;27:2957–63. 10.1093/bioinformatics/btr507 21903629 PMC3198573

[33] Li M , Tong H, Wang S et al. A chromosome-level genome assembly provides new insights into paternal genome elimination in the cotton mealybug *Phenacoccus solenopsis*. Mol Ecol Resou*r* 2020;20:1733–47. 10.1111/1755-0998.13232 33460249

[34] Li H , Durbin R. Fast and accurate short read alignment with Burrows-Wheeler transform. Bioinformatic*s* 2009;25:1754–60. 10.1093/bioinformatics/btp324 19451168 PMC2705234

[35] Danecek P , Bonfield JK, Liddle J et al. Twelve years of SAMtools and BCFtools. Gigascienc*e* 2021;10:giab008. 10.1093/gigascience/giab008 PMC7931819 33590861

[36] Morgulis A , Coulouris G, Raytselis Y et al. Database indexing for production MegaBLAST searches. Bioinformatic*s* 2008;24:1757–64. 10.1093/bioinformatics/btn322 18567917 PMC2696921

[37] Shen W , Le S, Li Y et al. SeqKit: a cross-platform and ultrafast toolkit for FASTA/Q file manipulation. PLoS On*e* 2016;11:e0163962. 10.1371/journal.pone.0163962 27706213 PMC5051824

[38] Wick RR , Judd LM, Gorrie CL et al. Unicycler: resolving bacterial genome assemblies from short and long sequencing reads. PLoS Comput Bio*l* 2017;13:e1005595. 10.1371/journal.pcbi.1005595 28594827 PMC5481147

[39] Aziz RK , Bartels D, Best AA et al. The RAST server: rapid annotations using subsystems technology. BMC Genomic*s* 2008;9:75. 10.1186/1471-2164-9-75 18261238 PMC2265698

[40] Krzywinski M , Schein J, Birol I et al. Circos: an information aesthetic for comparative genomics. Genome Re*s* 2009;19:1639–45. 10.1101/gr.092759.109 19541911 PMC2752132

[41] Huson DH , Albrecht B, Bagci C et al. MEGAN-LR: new algorithms allow accurate binning and easy interactive exploration of metagenomic long reads and contigs. Biol Direc*t* 2018;13:6. 10.1186/s13062-018-0208-7 29678199 PMC5910613

[42] Katoh K , Kuma K, Toh H et al. MAFFT version 5: improvement in accuracy of multiple sequence alignment. Nucleic Acids Re*s* 2005;33:511–8. 10.1093/nar/gki198 15661851 PMC548345

[43] Capella-Gutierrez S , Silla-Martinez JM, Gabaldon T. trimAl: a tool for automated alignment trimming in large-scale phylogenetic analyses. Bioinformatic*s* 2009;25:1972–3. 10.1093/bioinformatics/btp348 19505945 PMC2712344

[44] Nguyen LT , Schmidt HA, von Haeseler A et al. IQ-TREE: a fast and effective stochastic algorithm for estimating maximum-likelihood phylogenies. Mol Biol Evo*l* 2015;32:268–74. 10.1093/molbev/msu300 25371430 PMC4271533

[45] Yu G . Using ggtree to visualize data on tree-like structures. Curr Protoc Bioinformatic*s* 2020;69:e96. 10.1002/cpbi.96 32162851

[46] Stanke M , Steinkamp R, Waack S et al. AUGUSTUS: a web server for gene finding in eukaryotes. Nucleic Acids Re*s* 2004;32:W309–12. 10.1093/nar/gkh379 15215400 PMC441517

[47] Meng X , Ji Y. Modern computational techniques for the HMMER sequence analysis. ISRN Bioinfor*m* 2013;2013:252183, 1–13. 10.1155/2013/252183 25937944 PMC4393056

[48] Zhang Z , Li J, Zhao XQ et al. KaKs_Calculator: calculating Ka and Ks through model selection and model averaging. Genomics Proteomics Bioinformatic*s* 2006;4:259–63. 10.1016/S1672-0229(07)60007-2 17531802 PMC5054075

[49] Kanehisa M , Goto S. KEGG: kyoto encyclopedia of genes and genomes. Nucleic Acids Re*s* 2000;28:27–30. 10.1093/nar/28.1.27 10592173 PMC102409

[50] Kanehisa M , Sato Y, Kawashima M. KEGG mapping tools for uncovering hidden features in biological data. Protein Sc*i* 2022;31:47–53. 10.1002/pro.4172 34423492 PMC8740838

[51] Chen S , Zhou Y, Chen Y et al. fastp: an ultra-fast all-in-one FASTQ preprocessor. Bioinformatic*s* 2018;34:i884–90. 10.1093/bioinformatics/bty560 30423086 PMC6129281

[52] Kim D , Paggi JM, Park C et al. Graph-based genome alignment and genotyping with HISAT2 and HISAT-genotype. Nat Biotechno*l* 2019;37:907–15. 10.1038/s41587-019-0201-4 31375807 PMC7605509

[53] Pertea M , Pertea GM, Antonescu CM et al. StringTie enables improved reconstruction of a transcriptome from RNA-seq reads. Nat Biotechno*l* 2015;33:290–5. 10.1038/nbt.3122 25690850 PMC4643835

[54] Langfelder P , Horvath S. WGCNA: an R package for weighted correlation network analysis. BMC Bioinform 2008;9:559. 10.1186/1471-2105-9-559 PMC2631488 19114008

[55] Zhang B , Horvath S. A general framework for weighted gene co-expression network analysis. Stat Appl Genet Mol Bio*l* 2005;4:17. 10.2202/1544-6115.1128 16646834

[56] Wu T , Hu E, Xu S et al. clusterProfiler 4.0: a universal enrichment tool for interpreting omics data. Innovation 2021;2:100141. 10.1016/j.xinn.2021.100141 34557778 PMC8454663

[57] Livak KJ , Schmittgen TD. Analysis of relative gene expression data using real-time quantitative PCR and the 2(-delta delta C(T)) method. Method*s* 2001;25:402–8. 10.1006/meth.2001.1262 11846609

[58] Hosokawa T , Koga R, Kikuchi Y et al. *Wolbachia* as a bacteriocyte-associated nutritional mutualist. Proc Natl Acad Sci US*A* 2010;107:769–74. 10.1073/pnas.0911476107 20080750 PMC2818902

[59] Zhang Y , Zhang S, Xu L. The pivotal roles of gut microbiota in insect plant interactions for sustainable pest management. NPJ Biofilms Microbiome*s* 2023;9:66. 10.1038/s41522-023-00435-y 37735530 PMC10514296

[60] Ju JF , Bing XL, Zhao DS et al. *Wolbachia* supplement biotin and riboflavin to enhance reproduction in planthoppers. ISME *J* 2020;14:676–87. 10.1038/s41396-019-0559-9 31767943 PMC7031331

[61] Wang YB , Ren FR, Yao YL et al. Intracellular symbionts drive sex ratio in the whitefly by facilitating fertilization and provisioning of B vitamins. ISME *J* 2020;14:2923–35. 10.1038/s41396-020-0717-0 32690936 PMC7784916

[62] Yao YL , Ma XY, Wang TY et al. A bacteriocyte symbiont determines whitefly sex ratio by regulating mitochondrial function. Cell Re*p* 2023;42:112102. 10.1016/j.celrep.2023.112102 36774548

[63] Kono M , Koga R, Shimada M et al. Infection dynamics of coexisting beta- and gammaproteobacteria in the nested endosymbiotic system of mealybugs. Appl Environ Microbio*l* 2008;74:4175–84. 10.1128/AEM.00250-08 18469124 PMC2446506

[64] Masson F , Moné Y, Vigneron A et al. Weevil endosymbiont dynamics is associated with a clamping of immunity. BMC Genomic*s* 2015;16:1–13. 10.1186/s12864-015-2048-5 26482132 PMC4617454

[65] Moran NA , Bennett GM. The tiniest tiny genomes. Ann Rev Microbio*l* 2014;68:195–215. 10.1146/annurev-micro-091213-112901 24995872

[66] Garber AI , Kupper M, Laetsch DR et al. The evolution of interdependence in a four-way mealybug symbiosis. Genome Biol Evo*l* 2021;13:evab123. 10.1093/gbe/evab123 PMC8331144 34061185

[67] McCutcheon JP , von Dohlen CD. An interdependent metabolic patchwork in the nested symbiosis of mealybugs. Curr Bio*l* 2011;21:1366–72. 10.1016/j.cub.2011.06.051 21835622 PMC3169327

[68] Dedeine F , Vavre F, Fleury F et al. Removing symbiotic *Wolbachia* bacteria specifically inhibits oogenesis in a parasitic wasp. Proc Natl Acad Sci US*A* 2001;98:6247–52. 10.1073/pnas.101304298 11353833 PMC33453

[69] Lutwyche P , Cordeiro C, Wiseman DJ et al. Intracellular delivery and antibacterial activity of gentamicin encapsulated in pH-sensitive liposomes. Antimicrob Agents Chemothe*r* 1998;42:2511–20. 10.1128/AAC.42.10.2511 9756749 PMC105873

[70] Shan HW , Luan JB, Liu YQ et al. The inherited bacterial symbiont *Hamiltonella* influences the sex ratio of an insect host. Proc Biol Sc*i* 1915;286:20191677. 10.1098/rspb.2019.1677 PMC6892053 31744432

[71] Hou Y , Yao K, Yin Y et al. Endogenous synthesis of amino acids limits growth, lactation, and reproduction in animals. Adv Nut*r* 2016;7:331–42. 10.3945/an.115.010850 26980816 PMC4785480

[72] O'Brien DM , Fogel ML, Boggs CL. Renewable and nonrenewable resources: amino acid turnover and allocation to reproduction in Lepidoptera. Proc Natl Acad Sci US*A* 2002;99:4413–8. 10.1073/pnas.072346699 11930002 PMC123662

[73] Leitão-Gonçalves R , Carvalho-Santos Z, Francisco AP et al. Commensal bacteria and essential amino acids control food choice behavior and reproduction. PLoS Bio*l* 2017;15:e2000862. 10.1371/journal.pbio.2000862 28441450 PMC5404834

[74] Mossmann D , Park S, Hall MN. mTOR signalling and cellular metabolism are mutual determinants in cancer. Nat Rev Cance*r* 2018;18:744–57. 10.1038/s41568-018-0074-8 30425336

[75] Fenton TR , Gout IT. Functions and regulation of the 70kDa ribosomal S6 kinases. Int J Biochem Cell Bio*l* 2011;43:47–59. 10.1016/j.biocel.2010.09.018 20932932

[76] Stocker H , Radimerski T, Schindelholz B et al. Rheb is an essential regulator of S6K in controlling cell growth in *Drosophila*. Nat Cell Bio*l* 2003;5:559–66. 10.1038/ncb995 12766775

[77] Fidalgo da Silva E , Fong J, Roye-Azar A et al. Beyond protein synthesis; the multifaceted roles of tuberin in cell cycle regulation. *Front Cell* Dev Bio*l* 2021;9:806521. 10.3389/fcell.2021.806521 35096832 PMC8795880

[78] Jing J , Rui L, Junyuan S et al. Small-molecule compounds inhibiting S-phase kinase-associated protein 2: a review. Front Pharmaco*l* 2023;14:1122008. 10.3389/fphar.2023.1122008 37089937 PMC10113621

