# Supplementary material for: Endosymbiont Tremblaya phenacola influences the reproduction of cotton mealybugs by regulating the mechanistic target of rapamycin pathway
Source: ISME J. 2024 Mar 22;18(1):wrae052. doi: 10.1093/ismejo/wrae052 (PMC11014885; doi:10.1093/ismejo/wrae052)
Supplement: Supplementary_figure_wrae052 [file supplementary_figure_wrae052.docx]

# Supplementary Figures


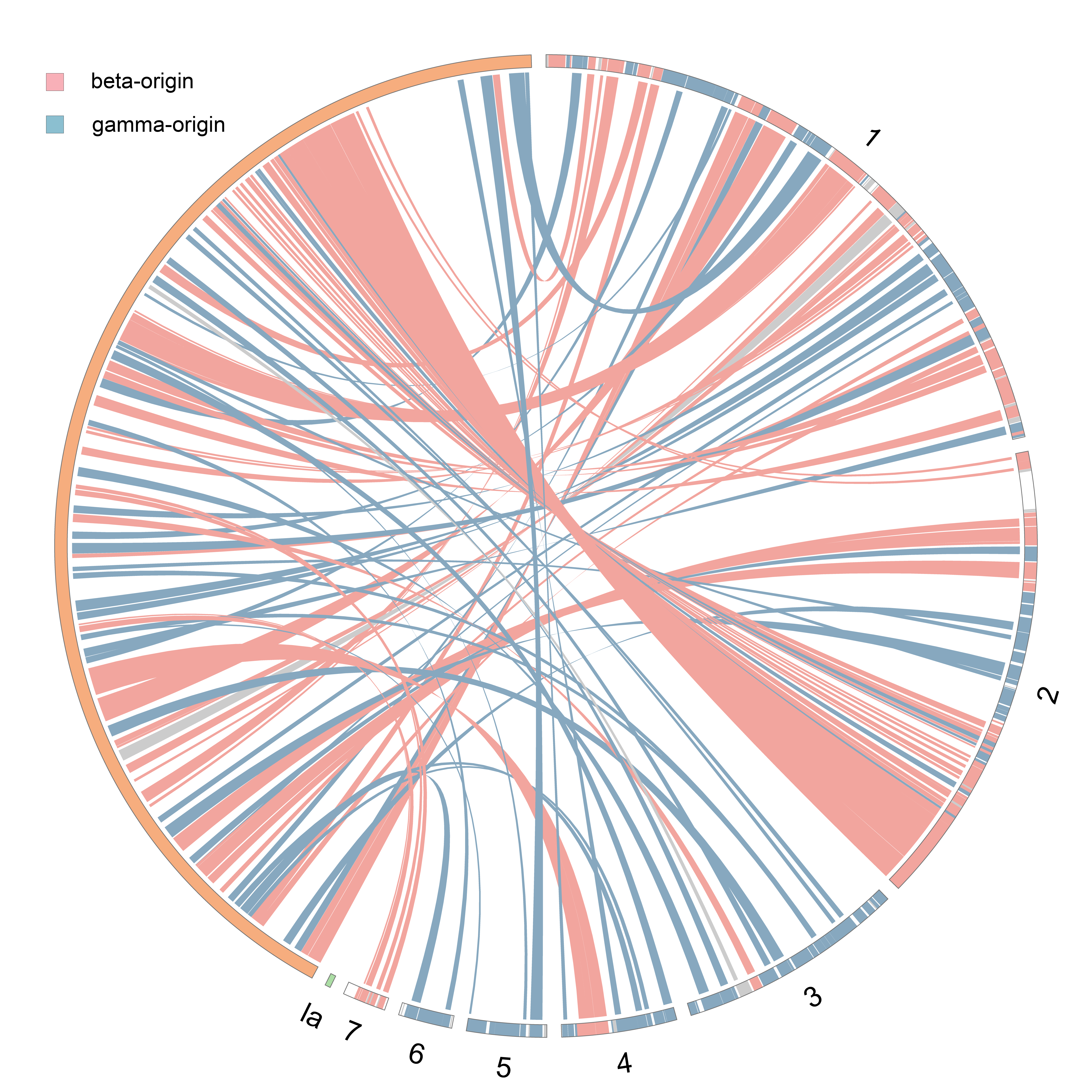


**Figure S1** Synteny blocks between *T. phenacola* PSOL and *T. phenacola* PAVE. 1-7 represent the contigs of *T. phenacola* PSOL.


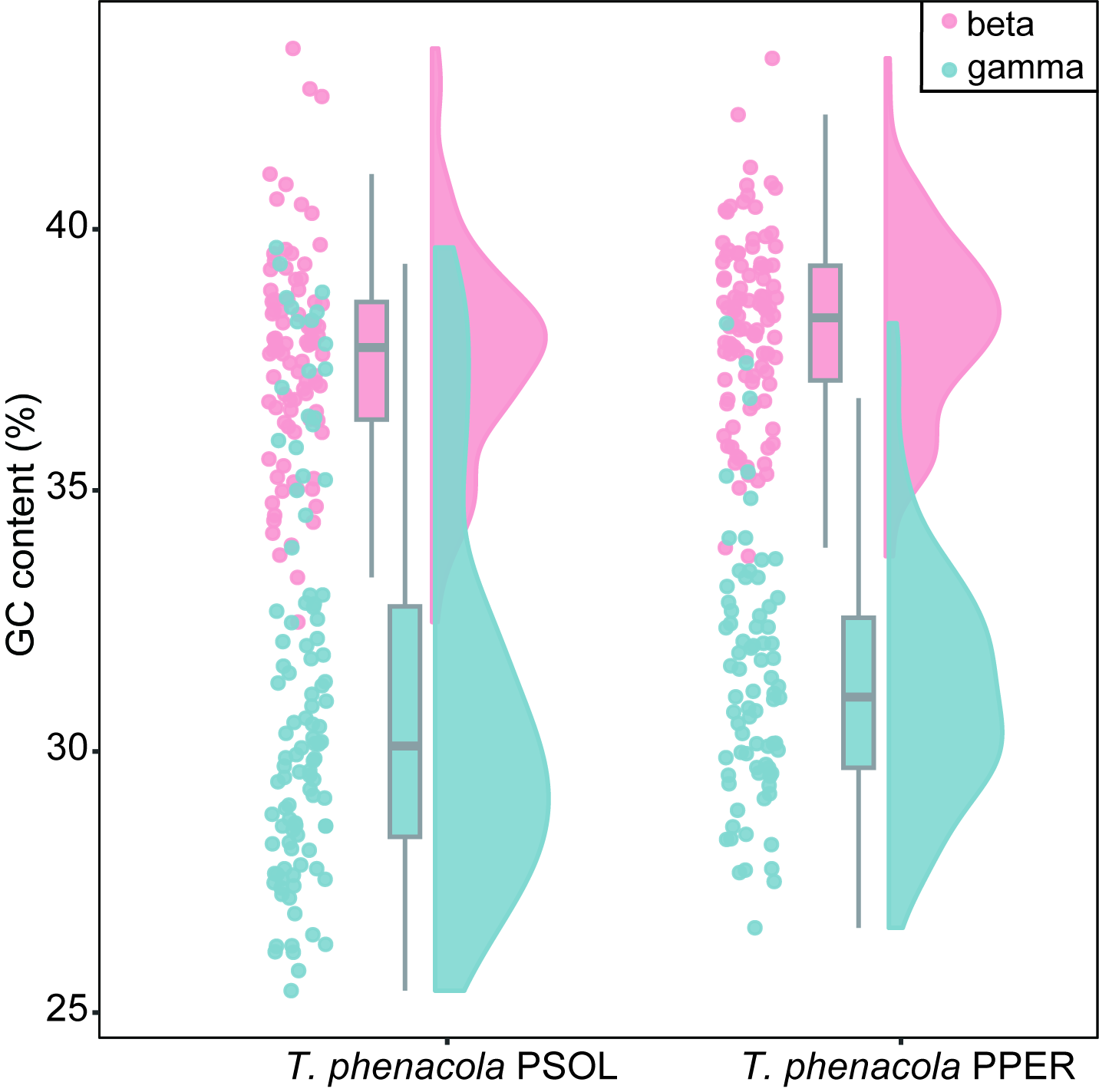


**Figure S2** GC content distribution of beta and gamma origin genes in *T. phenacola* PSOL and *T. phenacola* PPER


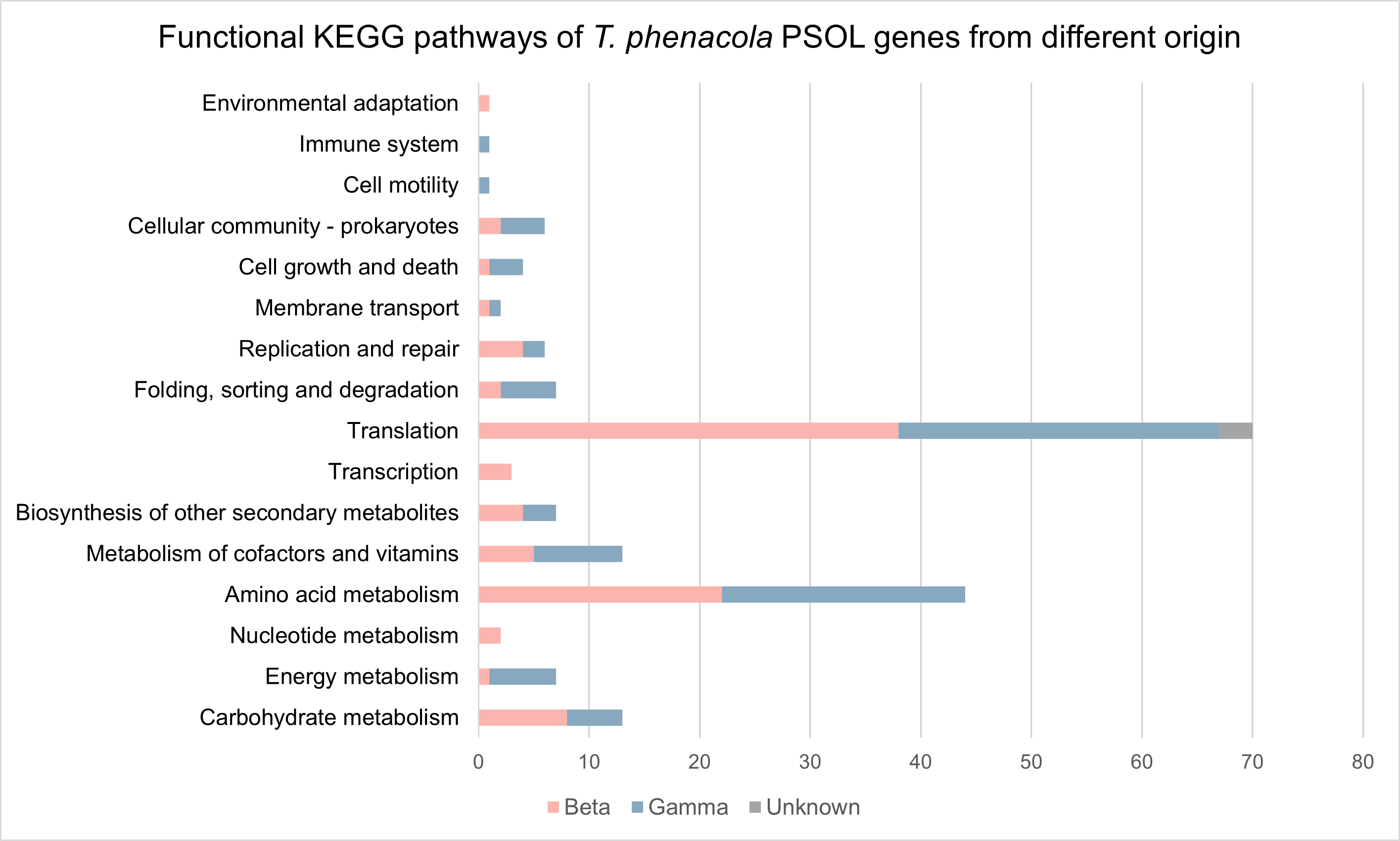
­

**Figure S3** Functional KEGG pathways of *T. phenacola* PSOL genes.


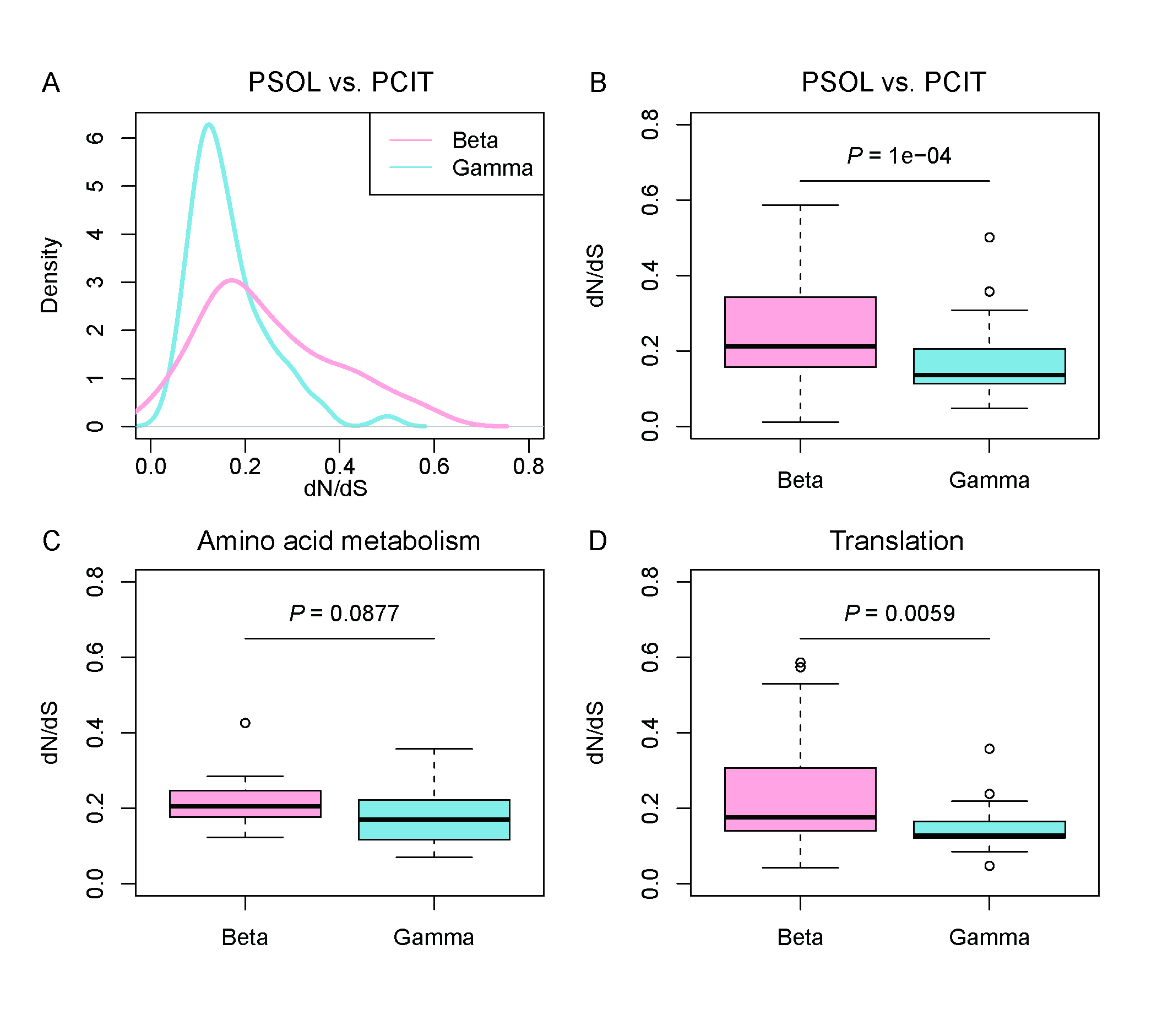


**Figure S4** Rates of synonymous (dS) and nonsynonymous (dN) substitutions between orthologous genes from the same lineage in *T. phenacola* PSOL and the symbiotic system of *T. princeps* PCIT and *Moranella endobia* PCIT. (A) Density distribution of dN/dS rates for beta-origin and gamma-origin genes. (B) dN/dS rates of beta-origin genes and gamma-origin genes (Wilcoxon rank sum test, *P* = 1e-4). (C) dN/dS rates of beta-origin genes involved in amino acid metabolism (*P* = 0.0877). (D) dN/dS rates of gamma-origin genes involved in translation (*P* = 0.0059).


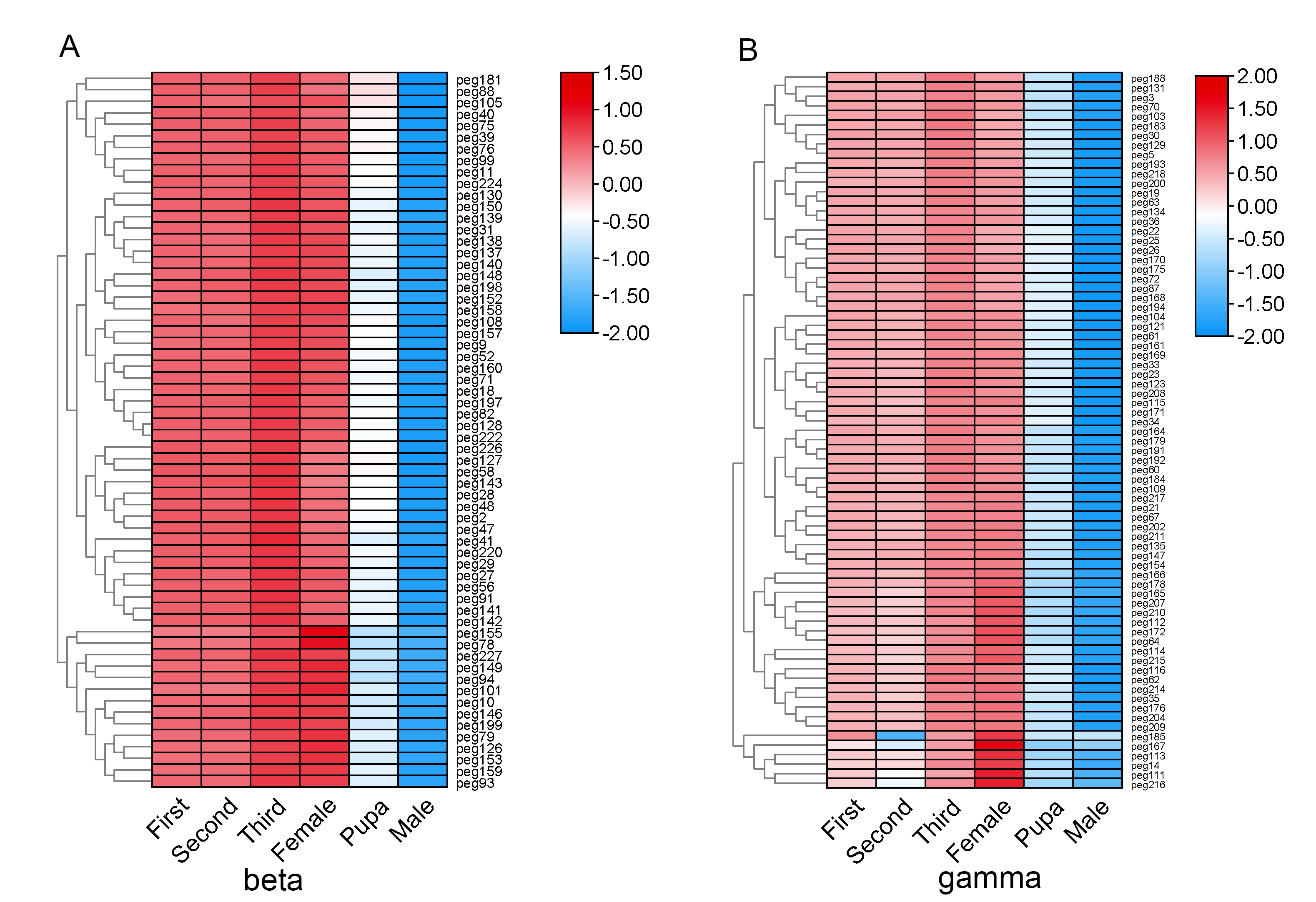


**Figure S5** The expression patterns of genes originating from beta (A) and gamma (B) in *T. phenacola* PSOL across various stages of host development.


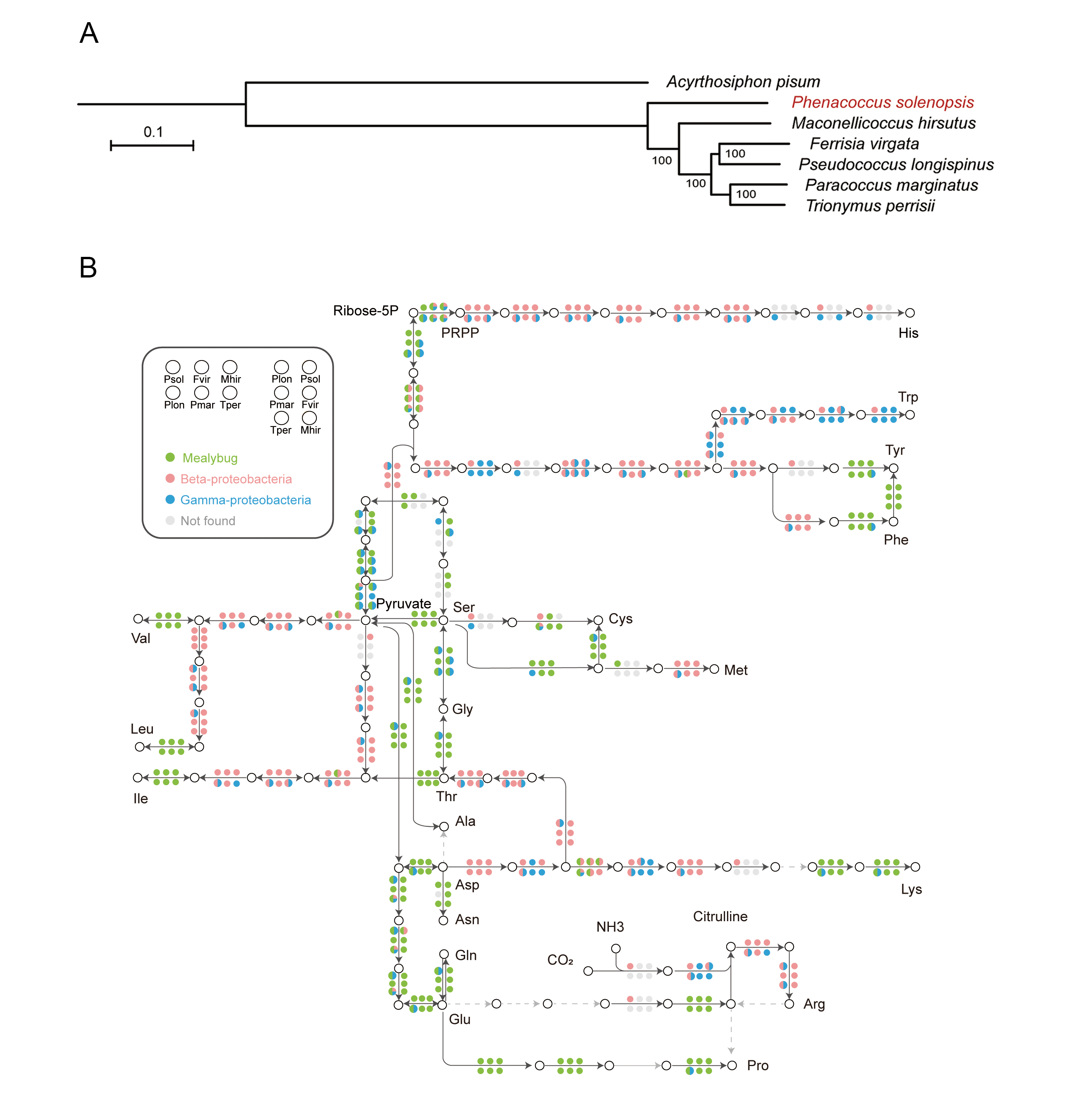


**Figure S6** Phylogenetic relationship (A) and amino acid synthesis pathways in six mealybugs (B). The six consecutive circles, both horizontal and vertical, correspond to the endosymbiotic systems of six distinct mealybug species. These species include *P. solenopsis* (Psol), *Ferrisia virgata* (Fvir), *Maconellicoccus hirsutus* (Mhir), *Pseudococcus longispinus* (Plon), *Paracoccus marginatus* (Pmar), *Trionymus perrisii* (Tper). The circles to which the arrows point symbolize amino acids or intermediate metabolic products. The green, pink, and blue circles indicate that this enzyme only exists in mealybugs, β-proteobacteria or γ-proteobacteria, respectively. The circles of multiple colors indicate that the enzyme is redundant in this system.


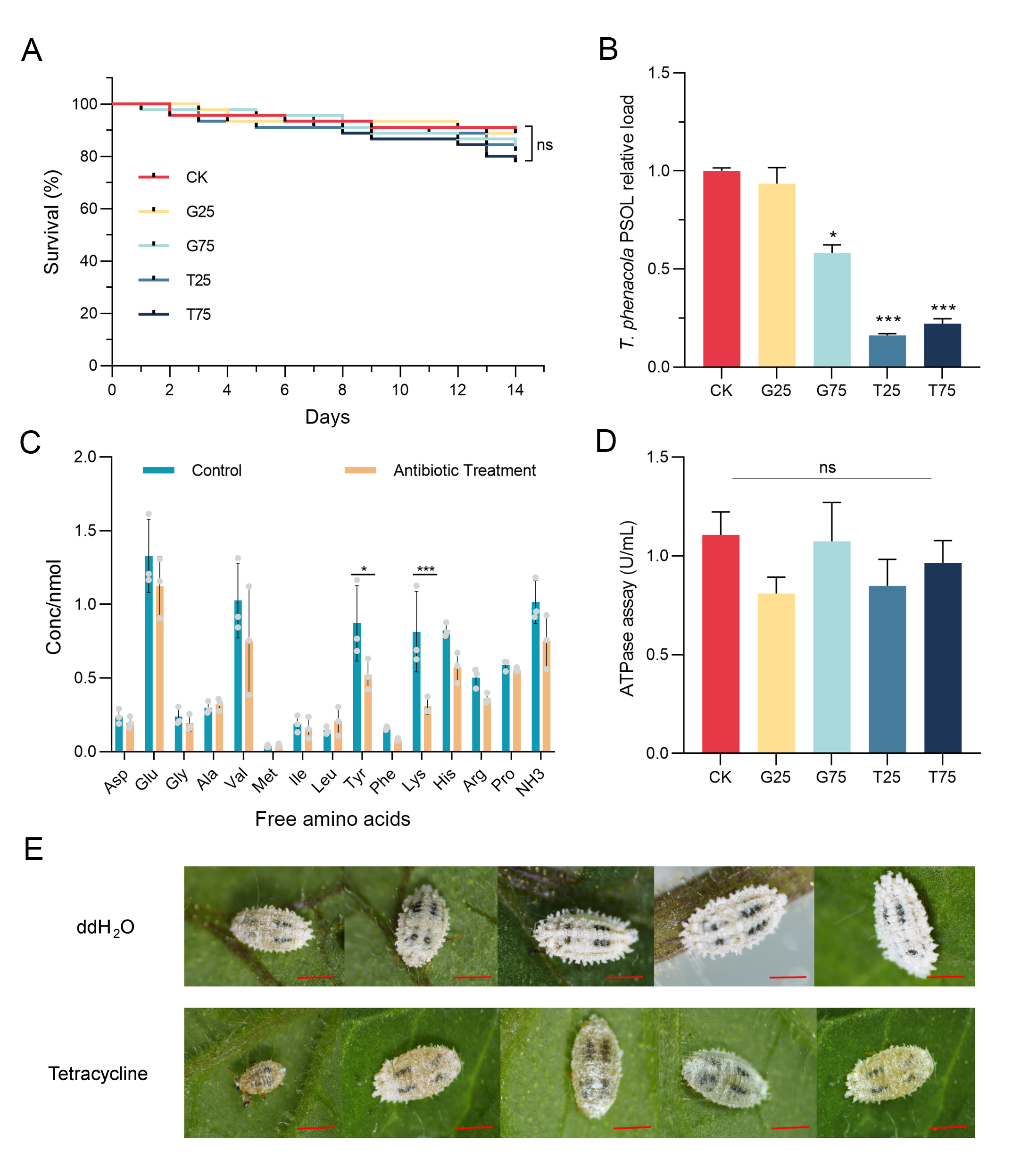


**Figure S7** The physiological impact of antibiotic treatment on the cotton mealybug. (A) The impact of different concentrations of gentamicin and tetracycline on the survival rate of mealybugs (n=45, log-rank test). (B) Relative quantification of *T. phenacola* PSOL in different groups (**P* < 0.05, *** *P* < 0.001). (C) The concentration of free amino acids in cotton mealybug after treated by 0.25 mg/ml tetracycline (**P* < 0.05, *** *P* < 0.001). (D) The assay of ATPase content in mealybugs treated with gentamicin and tetracycline. (E) Phenotypic images of mealybugs after treatment with tetracycline and ddH_2_O, and the red scale represents 1 mm. All experiments were independently replicated three times.


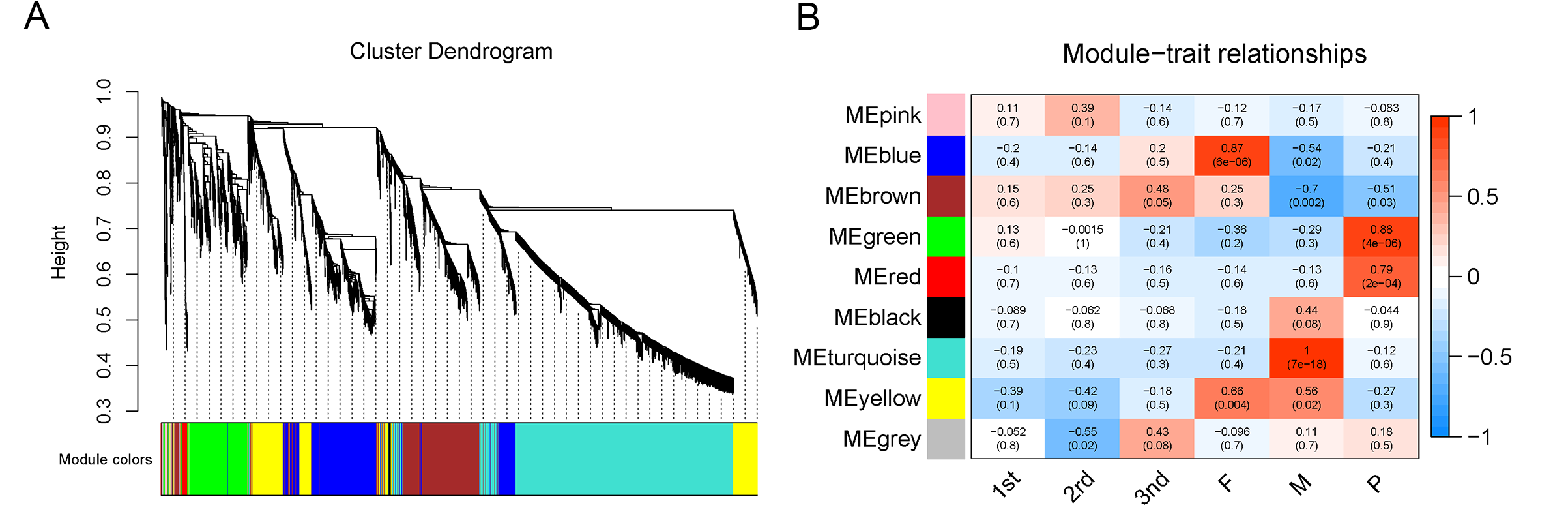


**Figure S8** Analysis of genes co-expression patterns between *P. solenopsis* and *T. phenacola* PSOL. (A) Clustering Pattern and module partition of genes expressed in the symbiotic system. (B) Correlation heatmap of different modules and different development stages of *P. solenopsis*. The numbers in parentheses represent *P* values. Numbers outside parentheses represent correlation coefficients.
